# Antioxidant Activity and Thermal Stability of Oleuropein and Related Phenolic Compounds of Olive Leaf Extract after Separation and Concentration by Salting-Out-Assisted Cloud Point Extraction

**DOI:** 10.3390/antiox3020229

**Published:** 2014-04-08

**Authors:** Konstantinos Stamatopoulos, Evangelos Katsoyannos, Arhontoula Chatzilazarou

**Affiliations:** 1 Department of Food Technology, Faculty of Food Technology and Nutrition, Technological Educational Institute of Athens, 12 Ag. Spyridonos St., Egaleo, Athens 12210, Greece; E-Mail: ekatso@teiath.gr; 2 Department of Oenology and Beverage Technology, Faculty of Food Technology and Nutrition, Technological Educational Institute of Athens, 12 Ag. Spyridonos St., Egaleo, Athens 12210, Greece; E-Mail: arhchatzi@yahoo.gr

**Keywords:** olive leaf extract, salting-out, cloud point extraction, polyphenols, Tween 80

## Abstract

A fast, clean, energy-saving, non-toxic method for the stabilization of the antioxidant activity and the improvement of the thermal stability of oleuropein and related phenolic compounds separated from olive leaf extract via salting-out-assisted cloud point extraction (CPE) was developed using Tween 80. The process was based on the decrease of the solubility of polyphenols and the lowering of the cloud point temperature of Tween 80 due to the presence of elevated amounts of sulfates (salting-out) and the separation from the bulk solution with centrifugation. The optimum conditions were chosen based on polyphenols recovery (%), phase volume ratio (V_s_/V_w_) and concentration factor (F_c_). The maximum recovery of polyphenols was in total 95.9%; V_s_/V_w_ was 0.075 and F_c_ was 15 at the following conditions: pH 2.6, ambient temperature (25 °C), 4% Tween 80 (w/v), 35% Na_2_SO_4_ (w/v) and a settling time of 5 min. The total recovery of oleuropein, hydroxytyrosol, luteolin-7-*O*-glucoside, verbascoside and apigenin-7-*O*-glucoside, at optimum conditions, was 99.8%, 93.0%, 87.6%, 99.3% and 100.0%, respectively. Polyphenolic compounds entrapped in the surfactant-rich phase (V_s_) showed higher thermal stability (activation energy (Ea) 23.8 kJ/mol) compared to non-entrapped ones (Ea 76.5 kJ/mol). The antioxidant activity of separated polyphenols remained unaffected as determined by the 1,1-diphenyl-2-picrylhydrazyl method.

## 1. Introduction

The health promoting properties of plant polyphenolic antioxidant compounds [1,2,3,4], as well as their potential application as natural food additives [5] have led to a great scientific and commercial interest. In addition, there is a consumer demand for food products free of artificial food additives, such as butylated hydroxytoluene (BHT) and butylated hydroxyanisole (BHA), since these chemically synthesized preservatives have been reported for carcinogenesis [6,7,8]. Consequently, a lot of effort has been expended on the extraction, isolation and separation of those natural secondary metabolites [9]. For this purpose, several extraction techniques (liquid-solid phase, liquid-liquid phase, supercritical fluid, accelerated pressurized, ultrasound and microwave-assisted extraction) have been developed [10]. Most of those methods are characterized either by the use of large solvent volumes and long extraction times or by high energy consumption and expensive facilities. Thus, difficulties in their application have emerged for analytical purposes or even more for the industrial production of natural phenolic antioxidants, especially for food applications [11]. Ethanol is the only organic solvent leading to a natural extract; although, costly ethanol recycling procedures by evaporation/condensation or distillation are required. In addition, solid phase extraction (SPE) shows a lower recovery of phenolic compounds, while supercritical fluid extraction (SFE) using liquid CO_2_ requires expensive, high-pressure equipment [12].

In contrast to the above-mentioned techniques, micelle-mediated cloud point extraction (CPE) has been recognized as a useful tool for the separation and pre-concentration of organic solutions [12]. CPE has been applied in the extraction or pre-concentration of metal ions [13], Polychlorinated biphenyl (PCBs), polycyclic aromatic hydrocarbons (PAHs), dichlorodiphenyltrichloroethane DDT, fungicides, pesticides, aromatic amines and fulvic and humic acids [14,15]. Katsoyannos *et al*. [16] and Gortzi *et al.* [17] have worked on the separation of polyphenols and tocopherols from olive mill wastewater (OMW), as well as carotenoids from red flesh orange with CPE. Non-ionic surfactants, which are mainly used in the CPE process, have high cloud points (50–100 °C). This fact limits the implementation of those surfactants, since the thermal degradation of polyphenols can occur.

CPE recoveries of 65.1% of polyphenols from OMW have been reported by Katsoyannos *et al.* [18] with the use of 5% surfactant in total, whereas a 98.5% recovery has been achieved when 20% surfactant (w/v) and NaCl 35% w/v were used. In addition, when a two-step CPE with a total of 4% v/v of Genapol X-080 or 10% v/v of polyethylene glycol PEG 8000 was applied in wine sludge, the phenol recovery values achieved were 75.8% or 98.5%, [19]. Nevertheless, a high amount of surfactant in the tested solution leads to low concentration factors (≈5), and hence, CPE may have some limitations for a sufficient pre-concentration of polyphenols, as well as for the mass production of natural antioxidants.

Several studies have shown the effect of electrolytes on the solubility of polyphenolic compounds (salting-out) [20,21,22]. However, this technique has been exclusively used for the removal of those phytochemicals from OMW in order to reduce the pollutant load of the wastes obtained by olive oil production. In addition, electrolytes can reduce significantly the cloud point of non-ionic surfactants to ambient temperatures [23], and hence, thermal degradation of natural antioxidant can be prevented, as well as energy can be saved by avoiding the heating of the tested solution.

The aim of the present work is to develop a salting-out-assisted cloud point extraction using Tween 80 as the food grade surfactant for the stabilization of the antioxidant activity and the improvement of the thermal stability of polyphenols from olive leaf extract. Optimization of the parameters affecting the recovery efficiency was performed, as well as the antiradical activity, and the thermal stability of the separated polyphenolic compounds was monitored.

## 2. Experimental Section

### 2.1. Materials

Methanol, acetic acid and acetonitrile were purchased from Merck (Darmstadt, Germany) and tyrosol and caffeic acid from Sigma-Aldrich (Hohenbrunn, Germany). Oleuropein, hydroxytyrosol, apigenin-7-*O*-glucoside, luteolin-7-*O*-glucoside and verbascoside were purchased from Extrasynthese (Genay, France), while sodium acetate trihydrate was from Carlo Ebra Reactifs-SDS (Val de Reuil Cedex, France). Rutin was purchased from Sigma (St. Louis, MO, USA). Sodium chloride was obtained from Panreac Química S.A. (Barcelona, Spain) and Tween 80 from Merck (Darmstadt, Germany). Sodium sulfate was from Chem-Lab NV (Zedelgem, Belgium) and ammonium sulfate from Merck (Darmstadt, Germany).

### 2.2. Extraction of Olive Leaves

Prior to extraction, olive leaves were processed according to Stamatopoulos, Katsoyannos, Chatzilazarou and Konteles [24]. Briefly, fresh olive leaves collected in October were steam blanched with a household steam cooker for 10 min at atmospheric pressure and then dried with a tray oven at 60 °C for 4 h and an air speed of 2 m/s. Subsequently, leaves were ground and sieved to a size of 0.3–1 mm and, finally, were extracted with water (solid-to-solvent ratio: 1:7). The extraction process was repeated twice, and the supernatants were collected and united after centrifugation (6000 rpm for 5 min).

### 2.3. Cloud Point Extraction Procedure

Olive leaf extract with a known concentration of oleuropein and related phenolic compounds was mixed with Tween 80. The prepared solution was then added to a plastic vial, which was followed by the addition of an amount of salt (NaCl, Na_2_SO_4_ or (NH_4_)_2_SO_4_) within the range of 0 (conventional CPE process with heating)–35% w/v. The solution containing the salt was then stirred vigorously with a vortex at room temperature until it became cloudy. The final solution was allowed to settle for a certain period of time (5–50 min). The effect of surfactant concentration on the efficiency of polyphenol separation was conducted within the range of 0.5%–11% (w/v) with a constant pH value (5.0 ± 0.2), salt (35%, w/v) and temperature (25 °C). Moreover, the optimum pH was examined within the range of 2.5–8.2 using either 0.1 M HCl or 0.1 M NaOH; at a constant Tween 80 concentration (4%, w/v), Na_2_SO_4_ concentration (35%, w/v) and temperature (25 °C). Qualitative and quantitative analysis of oleuropein and related phenolics was performed in the initial olive leaf extract, the surfactant-rich phase (V_s_) and the aqueous phase (V_w_) with high performance liquid chromatography with a diode array detector (HPLC-DAD). The recovery (%) was calculated from the initial concentration of the phenolic compounds (C_0_) in the solution before the separation (V_0_) and the concentration of the phenolics (C_w_) that remained in the aqueous phase (V_w_) after phase separation (Equation (1)):

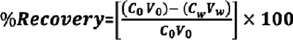
(1)


The concentration factor (F_c_) was calculated as follows:

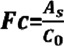
(2)
where:

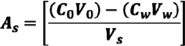
(3)


### 2.4. Thermal Stability of Polyphenols Entrapped in Surfactant-Rich Phase

The thermal stability of the entrapped polyphenols was investigated at several temperature intervals (70, 80, 100 °C). The surfactant phase was exposed at each temperature for 20, 40, 60, 80 and 100 min followed by cooling at 20 °C immediately after sampling. Subsequently, V_s_ was mixed with an equal volume of ethanol and was stirred vigorously with a vortex for 2 min. The mixture was centrifuged (6000 rpm, 3 min), and the supernatant was collected. The procedure was repeated 3 times. The ethanolic solution of the polyphenols was diluted 4 times prior to HPLC analysis. The impact of the thermal treatment on the polyphenolic compounds was evaluated by quantitative analysis of oleuropein, since it is the most abundant phenolic compound present in olive leaf extract. The results were used to plot lnC (C: oleuropein concentration) *vs.* time (min) at each temperature. Degradation rate constant k (min^−1^) was determined by the slope of each curve (lnC *vs.* time). Subsequently, the Arrhenius equation was used for the determination of activation energy (Ea, kJ/mol). The thermal degradation rate and the activation energy of entrapped polyphenols (V_s_) were compared to non-entrapped ones (extract).

### 2.5. Chromatographic Conditions

The equipment used was a HITACHI coupled to an autosampler L-2200, a pump L-2130, a column oven L-2300 and a diode array detector L-2455 and controlled by Agilent EZChrom Elite software. The column was a Pinnacle II RP C_18_, 3 μm, 150 × 4.6 mm (Restek), protected by a Kromasil 100–5–C18 guard cartridge starter kit for a 3.0/4.6 mm id. The column oven was set at 40 °C. Eluent (A) and (B) were 0.02 M sodium acetate adjusted at pH = 2.8 with acetic acid and pure acetonitrile, respectively. The flow rate was 1 mL/min. The elution gradient profile was as follows: starting (A), 100%; 2 min, 98%; 7 min, 95%; 16 min, 86%; 23 min, 82%; 30 min, 60%. The elution was monitored at 280 nm for oleuropein, hydroxytyrosol and tyrosol, at 330 nm for verbascoside and at 355 nm for luteolin and apigenin glucosides.

#### Calibration Curves of Oleuropein, Verbascoside, Luteolin-7-*O*-Glucoside Apigenin-7-*O*-Glucoside and Hydroxytyrosol

Ethanolic stock solutions were prepared for oleuropein, luteolin-7-O-glucoside, apigenin-7-O-glucoside, hydroxytyrosol and verbascoside in the range of 2–2000 ppm, 11–300 ppm, 8–200 ppm and 50–900 ppm, respectively. All the solutions were filtered through 0.45-μm syringe filters:

Oleuropein: *y* = 21,062*x* + 463,357; *R*^2^ = 0.9985

Luteolin-7-*O*-glucoside: *y* = 225,917*x* − 536,945; *R*^2^ = 0.9996

Apigenin-7-*O*-glucoside: *y* = 104,154*x* + 965,557; *R*^2^ = 0.9988

Verbascoside: *y* = 81,434*x* + 107,304; *R*^2^ = 0.9998

Hydroxytyrosol: *y* = 69,846*x* + 289,215; *R*^2^ = 0.9994

### 2.6. Determination of Antioxidant Activity

Antiradical activity (AA) was performed using the 2,2,-diphenyl-2-picryl-hydrazyl (DPPH) assay according to Braca *et al.* [25], with some modifications. Briefly, 2.5 mg of DPPH powder were diluted in 100 mL pure methanol with an absorption of 0.7 (±0.03) at 517 nm. The initial olive leaf extract was diluted 50 times with distilled water and then directly added to the DPPH solution. An aliquot of 1 mL of 0.004% DPPH solution was added in a cuvette with 33 μL of the diluted sample. As a control, 33 μL of distilled water were added instead of olive leaf extract. In addition, the antioxidant activity of separated phenolic compounds was determined by the extracting of V_s_ with an equal volume of ethanol and mixing vigorously with a vortex for 1 min. Subsequently, the mixture was diluted 84 times with ethanol, and then, 33 μL of the sample were added to 1 mL of the DPPH solution. The reaction mixtures were vortex-mixed and were allowed to stand in the dark for 30 min at room temperature before measuring the decrease in absorbance at 517 nm. As a control, 33 μL of ethanol were directly added to the DPPH solution. The spectrophotometer (SHIMADZU mini 1240 UV-Vis, Shimadzu, Columbia, MD, USA) was calibrated with pure methanol. Antioxidant activity was expressed as the percentage of inhibition of the DPPH radical and was calculated by the following Equation (4):

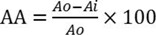
(4)
where *A*_0_ and *A*_i_ stand for the absorbance of the control sample and the sample containing olive leaf extract, respectively.

### 2.7. Statistical Analysis

All determinations were carried out at least in triplicate, and the values were averaged and given along with the standard deviation (±SD). For all statistical work, Microsoft Excel™ 2010 was used.

## 3. Results and Discussion

The aim of this work was to develop a salting-out-assisted cloud point extraction for a sufficient separation of oleuropein and related phenolics from olive leaf extract in a single CPE step, as well as monitoring the antioxidant activity and thermal stability of these nutraceuticals entrapped in the surfactant-rich phase.

### 3.1. Salting-Out-Assisted Cloud Point Procedure

#### 3.1.1. Effect of the Addition of Salt

**Figure 1:**
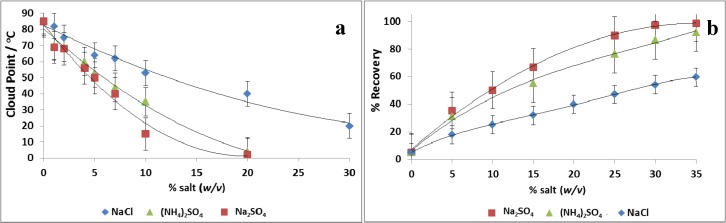
The influence of added salts (**a**) in the phase separation of a mixture of olive leaf extract (total phenols concentration: 2500 ppm) and 4% Tween 80 (w/v) (pH 5 ± 0.2) and (**b**) in the percent of recovery of total phenolics from olive leaf extract; conditions: 4% Tween 80 (w/v), pH 5.0 ± 0.2, settling time 30 min. The total phenol concentration (ppm) is the sum of the concentration of oleuropein, verbascoside, luteolin-*O*-7-glucoside, apigenin-*O*-7-glucoside and hydroxytyrosol in the olive leaf extract, which were quantitated with an HPLC-diode array detector (DAD).

The depression of the cloud point of 4% Tween 80 (w/v) in olive leaf extract was investigated with the addition of NaCl, Na_2_SO_4_ and (NH_4_)_2_SO_4_ at several intervals. Figure 1a shows that Na_2_SO_4_ can effectively decrease the cloud point of Tween 80 (86 °C in the absence of electrolytes). The addition of 10% of Na_2_SO_4_ (w/v) or more decreases the cloud point below the normal ambient temperatures (*i.e.*, 25–30 °C). Hence, CPE can be operated without heating up the olive leaf extract at elevated temperatures, which could lead to the thermal degradation of the phenolic compounds. Ammonium sulfate ((NH_4_)_2_SO_4_) gave relatively similar results, whereas more than 25% w/v of NaCl were necessary to depress the cloud point temperature of Tween 80 below 35 °C. This behavior follows the order of the Hofmeister series [26]. Additionally, Nishi *et al.* reported that SO_4_^2−^ depressed the cloud point of non-ionic surfactant more effectively than Cl^−^ [27].

Subsequently, the effect of the addition of salts on the recovery of oleuropein and related phenolics (2500 ppm in initial olive leaf extract) was investigated by adding 4% w/v Tween 80, 35% salt (w/v) at pH 5.0 ± 0.2 (pH of olive leaf extract). The settling time was 30 min at room temperature. Clouding was observed for the solutions with all salts, NaCl, Na_2_SO_4_ and (NH_4_)_2_SO_4_. The surfactant-rich phase (containing phenolics) floated to the upper surface after settling for 30 min. Figure 1b illustrates the effectiveness of applying salting-out-assisted cloud point extraction with the use of Na_2_SO_4_ at elevated amounts of 30%–35% (w/v). Noubigh *et al.* [21] showed that Na_2_SO_4_ has a salting-out effect on the phenolic compounds, which increases as the salt concentration increases. Notably, the addition of sulfate salts provided a higher recovery for polyphenols than the chloride salt. However the addition of Na_2_SO_4_ gave a 6.6% higher recovery (96.4%) compared to (NH_4_)_2_SO_4_ (89.8%) (Figure 1b). The recovery that was reached with the proposed method is even higher than the value (94.4%) that obtained by Katsoyannos *et al.* [18] after double CPE in oil mill wastewater (OMW) using, in total, 10% Tween 80 (w/v) and 20% NaCl (w/v). It should be pointed out that based on the conventional CPE procedure (heating the solution above the cloud point of the surfactant), the recovery of polyphenols was only 5% (Figure 1a; the percent of recovery at Point 0 of the *x*-axis). Thus, salting-out-assisted cloud point extraction using Na_2_SO_4_ seems to improve the recovery of polyphenols remarkably, and hence, Na_2_SO_4_ was the proper candidate for subsequent experiments.

#### 3.1.2. Effect of the pH of the Solution

The influence of the pH on the recoveries of phenolic compounds present in olive leaf extract was evaluated. In this case, the experiments were performed with 4% Tween 80 adjusted to pH 2–8.2, whereas 35% Na_2_SO_4_ (w/v) was added to the solution.

It is well known that pH plays a significant role in the interaction of polyphenols with other constitutes, such as proteins [28]. Thus, salting-out-assisted cloud point extraction was conducted with and without filtrated (0.1 μm) olive leaf extract in order to investigate any interference in the separation of polyphenols by macromolecules.

**Figure 2:**
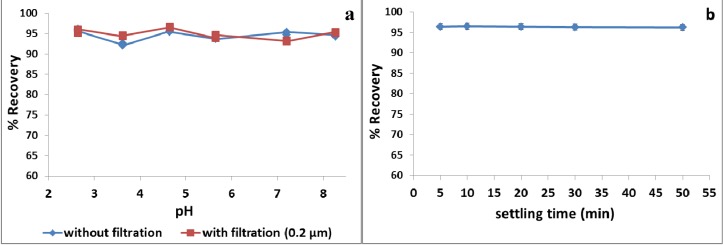
(**a**) The percent of recovery of the total phenols from olive leaf extract as a function of solution pH (settling time 30 min) and (**b**) the percent of recovery of polyphenols from olive leaf extract as a function of the settling time. Conditions: total polyphenols concentration: 2500 ppm; 4% Tween 80 (w/v); 35% Na_2_SO_4_ (w/v). The total phenol concentration (ppm) is the sum of the concentration of oleuropein, verbascoside, luteolin-*O*-7-glucoside, apigenin-*O*-7-glucoside and hydroxytyrosol in the olive leaf extract, which were quantitated with HPLC-DAD.

As can be seen in Figure 2a, the maximum recovery of polyphenols was obtained at pH 2.6 with 96.62% ± 0.27% and 95.34% ± 0.43% for the non-filtrated and filtrated samples, respectively. In addition, there were no significant differences in the recovery of polyphenols within the entire pH scale (*i.e.*, pH = 8.2; 95.61 ± 0.21% for filtrated sample and 94.91 ± 0.19% for non-filtrated one).

#### 3.1.3. Effect of Settling Time

The influence of the settling time on the recovery of polyphenols from olive leaf extract was also investigated. After adding Na_2_SO_4_ (35%, w/v) to a 4% Tween 80 (w/v)/olive leaf extract (total phenolics: 2500 ppm) solution and mixing with a vortex for 2 min, the liquid was left to stand at room temperature (25 °C) for varying lengths of time. Polyphenols were quantitatively extracted into the surfactant-rich phase after settling for 5 min (Figure 2b). Beside the fact that no significant differences were observed in the recovery of polyphenols for the entire settling time scale, the surfactant-rich phase obtained after only 5 min, however, was unstable and easily broken. The settling time value of 10 min was enough for the stable and complete separation of the surfactant-rich phase. Nevertheless, a recovery of polyphenols as high as 96.2% ± 1.4% was achievable after mixing the solution with a vortex for 2 min, settling the sample for 5 min and subsequently centrifuging it (3 min, 6000 rpm). Therefore, a short settling time and the acceleration of the phase separation by centrifugation were followed in the subsequent experiments.

#### 3.1.4. Effect of Surfactant Concentration

**Figure 3:**
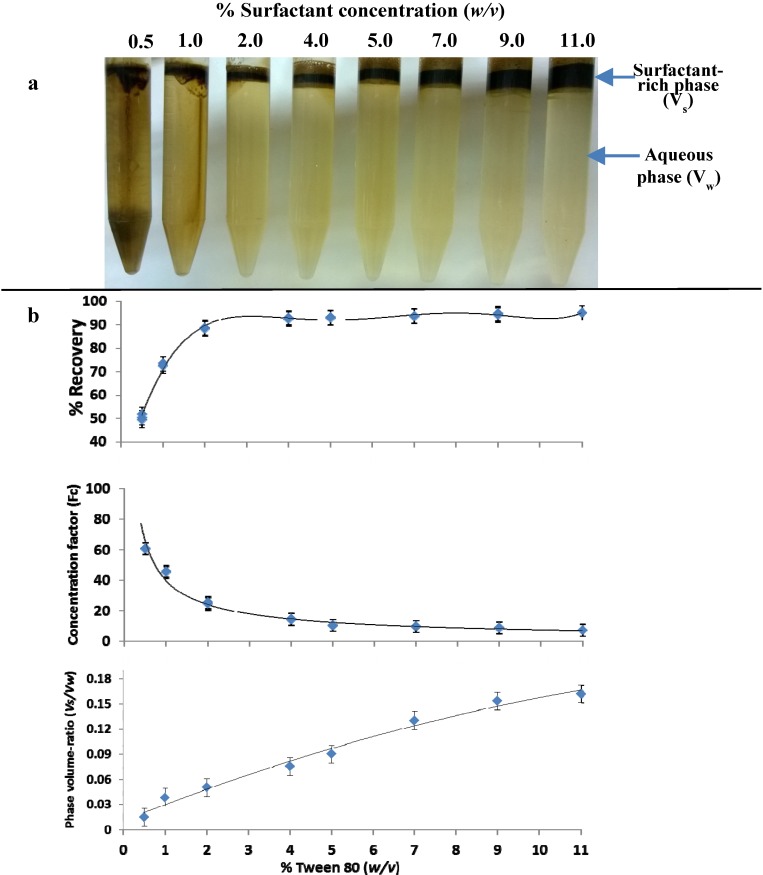
The influence of the added Tween 80 quantity in the performance of salting-out cloud point extraction (CPE). (**a**) Photographs of the phase separations and (**b**) the percent of recovery efficiency, concentration factor (F_c_) and phase volume-ratio (V_s_/V_w_) of salting-out-assisted CPE of polyphenols from olive leaf extract (2500 ppm). The amount of Na_2_SO_4_ added to olive leaf extract was 35% (w/v); pH: 2.6; settling time: 5 min; liquid temperature: 25 °C.

**Figure 4:**
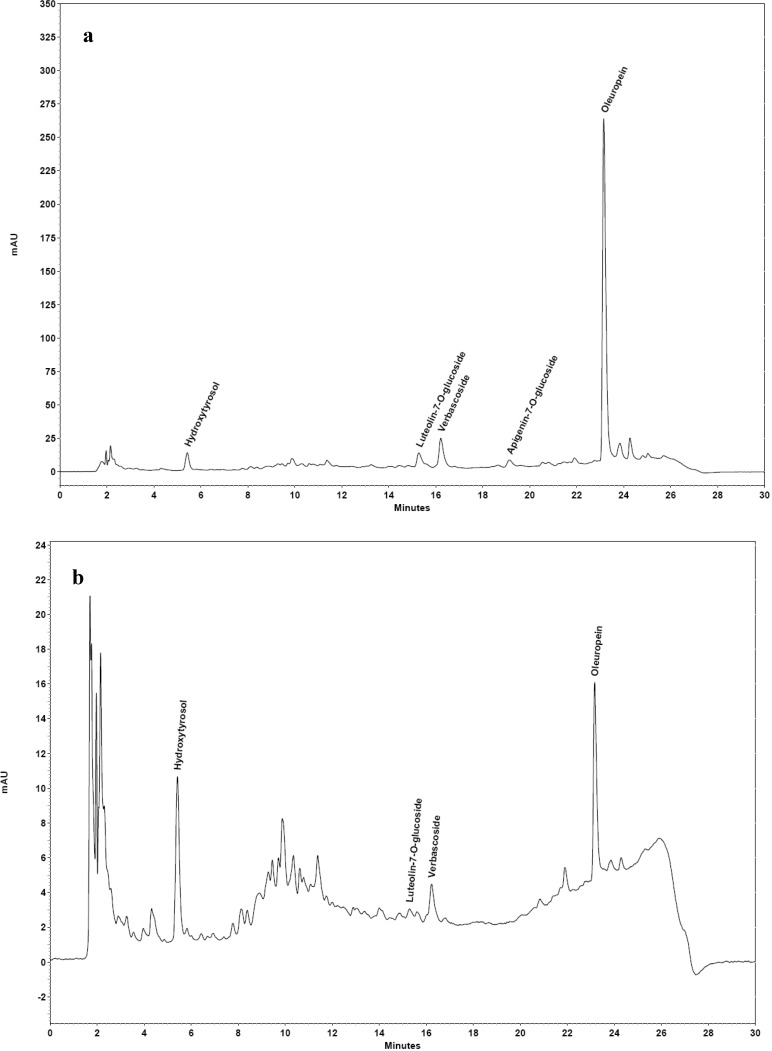
Chromatographic profile at 280 nm of the initial olive leaf extract (**a**) and the water phase (V_w_) after single-stage salting-out-assisted CPE (**b**). The samples were diluted two times prior to HPLC analysis.

The effect of the concentration of Tween 80 (0.5%–11%, w/v) in the extraction solution on the separation of polyphenols from olive leaf extract was investigated. A quantitative extraction of phenolic compounds was achieved when the Tween 80 concentration in solutions was 0.5% (Figure 3b). It should be noted that part of the polyphenols were precipitated for solutions with a Tween 80 concentration <2%, whereas, the surfactant floated to the upper surface after centrifugation (Figure 3a). Thus, estimating the recovery of polyphenols with Equation (1) and the concentration factor (F_c_), which includes Equation (3), led to an overestimation of the efficiency of salting-out-assisted CPE. Although, the concentration of polyphenols in the aqueous phase (V_w_) was decreased, this was not due to the entrapment of polyphenols in the micelles of Tween 80, but due to the decrease of their solubility and, hence, precipitation by the presence of Na_2_SO_4_. Thus, the efficiency of the method was evaluated with the determination of polyphenols concentration (C_s_) in V_s_, dividing this value with the initial concentration (C_0_) and expressing it as the percent of recovery. Figure 3 shows that the recovery of polyphenols was increased within a range of 0.5%–2% Tween 80 (w/v) and reached a plateau between 4% and 11% Tween 80 (w/v). Thus, it is advisable to use the lowest amount of Tween 80 (*i.e.*, 4%) in order to obtain the lowest phase volume-ratio (V_s_/V_w_) and, hence, the highest concentration factor (F_c_). The total recovery of polyphenols with 4% Tween 80 (w/v), 35% Na_2_SO_4_, pH 2.6 at ambient temperature, was 95.9 ± 1.2%, whereas F_c_ was 16 and V_s_/V_w_ was 0.075. This means that 1 L of olive leaf extract, which contains 2.5 g of polyphenols (*i.e.*, 2500 ppm), was concentrated in a volume of 75 mL. Figure 4 shows the chromatograms of the initial olive leaf extract (Figure 4a) and the V_w_ (Figure 4b).

#### 3.1.5. Effect of Temperature

The influence of the liquid temperature on the recovery of polyphenols was also investigated. After mixing 4% Tween 80 (w/v) with olive leaf extract (total phenols: 2500 ppm), 35% Na_2_SO_4_ (w/v) was added. While stirring the solution, the liquid temperature was varied from four to 60 °C using either a refrigerated bath or a thermostatic bath. In the temperature range from four to 10 °C, crystals of Na_2_SO_4_ appeared in the solution, and the phase separation was not complete, even after settling for 1 h. It should be pointed out that for temperatures from 45 to 60 °C, the phase separation was instantaneous; however, no significant differences were observed on the recovery of polyphenols compared to the values that were obtained at 20–35 °C.

Table 1 shows the concentrations of the individual phenolic compounds present in olive leaf extract and in V_w_ as well as their recoveries obtained at optimum conditions of CPE.

**Table 1:** Recoveries of the main polyphenolic compounds detected in the initial olive leaf extract and the water phase (V_w_) of single salting-out-assisted CPE in the presence of 35% Na_2_SO_4_ (w/v). ^1^C_o_: the concentration of phenolic compounds present in the initial olive leaf extract. The sample was diluted four times prior to HPLC analysis; ^2^C_w_: the concentration of phenolic compounds present in the aqueous phase after salting-out CPE; ND: not detected; ±standard deviation (*n* = 3). Conditions: pH: 2.6; 4% Tween 80 (w/v); settling time: 5 min; liquid temperature: 25 °C.

Analytes	Concentration (ppm)		% Recovery
	^1^C_0_	^2^C_w_	
Oleuropein	527.5 ± 2.30	1.06 ± 0.32	99.8 ± 0.13
Hydroxytyrosol	83.4 ± 0.41	5.83 ± 0.41	93.0 ± 0.19
Verbascoside	22.9 ± 0.12	0.16 ± 0.01	99.3 ± 0.31
Luteolin-*O*-7-glucoside	8.8 ± 0.64	1.07 ± 0.06	87.6 ± 1.10
Apigenin-*O*-7-glucoside	8.6 ± 0.22	ND	100.0

#### 3.1.6. Precision of the Method

In order to know the precision of the salting-out-assisted CPE method, reproducibility and repeatability within-laboratory were evaluated in a single experimental set-up with triplicates at the optimum conditions (Section 3.1.6). The results obtained are listed in Table 2 for each phenolic compound. The repeatability, expressed as the relative standard deviation, was from 3.95% to 6.14%; meanwhile, within-laboratory reproducibility ranged from 8.58% to 10.55%.

**Table 2:** Results obtained from the evaluation of the precision of the salting-out-assisted CPE method in terms of the repeatability relative standard deviation (s_r_) and the within-laboratory reproducibility relative standard deviation (s_WR_) for each phenolic compound.

Analyte	s_r_ (%)	s_WR_ (%)
Oleuropein	0.44	3.36
Hydroxytyrosol	0.51	4.05
Verbascoside	0.52	3.45
Luteolin-*O*-7-glucoside	7.27	3.00
Apigenin-*O*-7-glucoside	2.56	3.27

### 3.2. Thermal Stability of Polyphenols Entrapped in Surfactant-Rich Phase

**Figure 5:**
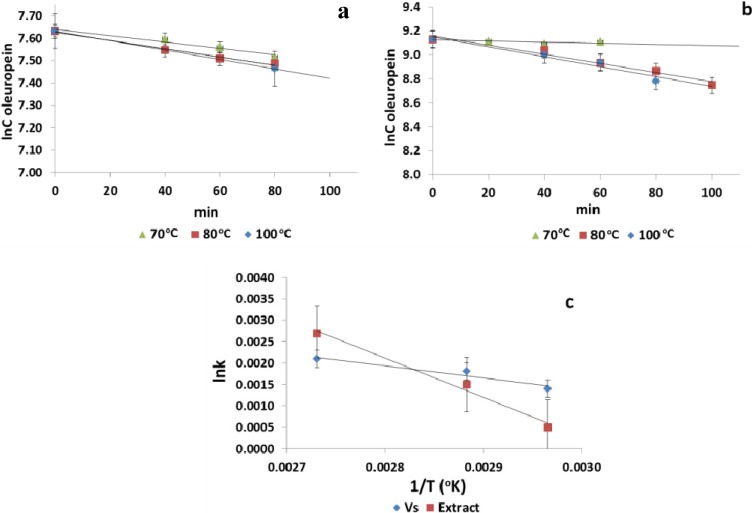
First-order kinetic curves of thermal degradation of (**a**) oleuropein entrapped in the surfactant-rich phase and (**b**) oleuropein present in the olive leaf extract; (**c**) lnk *vs.* 1/T kinetic curves for the thermal degradation of non-entrapped and entrapped oleuropein.

The thermal stability of polyphenolic compounds from olive leaf extract entrapped in V_s_ by salting-out-assisted CPE was investigated and compared to non-entrapped ones. For this reason, the surfactant phase, as well as the olive leaf extract were exposed to different temperatures for various time intervals (Figure 5). In this way, the activation energy values were determined by the Arrhenius equation based on the oleuropein degradation rate, since it is the most abundant polyphenolic compound in olive leaf extract and, consequently, in the surfactant-rich phase. The rate constants (k, min^−1^) of the thermal degradation of oleuropein at 70, 80 and 100 °C were 0.0005, 0.0038 and 0.0041 min^−1^ for the extract (Figure 5b) and 0.0014, 0.0018 and 0.0021 min^−1^ for V_s_ (Figure 5a). The results indicate that the thermal degradation of oleuropein occurred at a faster rate in the extract than in V_s_. Degradation was primarily caused by oxidation, cleavage of covalent bonds or enhanced oxidation reactions, due to thermal processing [29]. V_s_ contents only 0.04% water compared to 99.91% in the olive leaf extract. Thus, an explanation for the low degradation rates of oleuropein entrapped in Tween 80 might be the low moisture content, since water acts as a heat conductor and dilutor of the compounds, which is necessary in degradation reactions. Another reason could be the lower diluted oxygen in the surfactant-rich phase compared to the olive leaf extract.

Data on activation energy values found in the literature were mainly for individual anthocyanins and total anthocyanins present in juices, concentrates or extracts of fruits and vegetables, varying, in general, between 37.5 and 91.1 kJ/mol [30]. Regarding energy activation values (Figure 5c), our results indicate the ability of salting-out-assisted CPE to improve the heat sensitivity of entrapped oleuropein (Ea = 23.8 ± 1.5 kJ/mol) compared to the free one (Ea 76.5 ± 3.7 kJ/mol) in the olive leaf extract. That means that low activation energy implies that a higher temperature change is needed to degrade a specific compound more rapidly [29]. The Ea of oleuropein in the extract is higher compared to the values of individual phenolic compounds reported in the literature by Kopjar *et al.* [30]. Briante *et al.* [31] found no qualitative degradation of oleuropein at 40, 50, 60, 70 °C for a 3-h thermal treatment after TLC analysis. Similarly, in our thermal degradation kinetic analysis, neither qualitative nor quantitative (HPLC analysis) changes were observed when oleuropein was treated at 70 °C for 1 h; although, this is not the case when olive leaf extract is treated at higher temperatures.

### 3.3. Antioxidant Activity (DPPH) of the Recovered Polyphenolic Compounds

Antioxidant activity can be a suitable marker in order to evaluate alternations in the bioactivity of polyphenols by the salting-out CPE process. Studies have shown that encapsulation of polyphenols with different materials (e.g., maltodextrins, cyclodextrins, modified starch and chitosan) retains their antioxidant activity and protects them from oxidation [32]. After the recovery of polyphenols from V_s_ with ethanol, the DPPH assay was used for the determination of the antioxidant activity of phenolic compounds. The ethanolic solution was diluted 84 times in order to have the same total phenol content as the diluted olive leaf extract (*i.e.*, 29.7 ppm). Both solutions showed relatively similar inhibition values: 60.8% ± 1.2% (extract) and 62.1% ± 1.7% (V_s_). Chromatographic analysis showed that the solution of the recovered polyphenols from Tween 80 had the same phenolic profile as the olive leaf extract. Thus, the same mixture of phenolic compounds contributes to the overall antioxidant activity of both solutions. In conclusion, the separation of polyphenol compounds by the salting-out CPE procedure using Tween 80 as the surfactant could be a potential method for the production of natural antioxidants without affecting the bioactivity of polyphenols.

## 4. Conclusions

Micellar-mediated systems have been used by several researchers in the separation of organic compounds, vitamin K, E and A [33,34], as well as carotenoids, tocopherols and phenolic compounds [18].

The current study showed that salting-out-assisted CPE, in contrast to previous methods [16,17,18,19], remarkably improves the separation of the polyphenols present in olive leaf extract using sulfates. Satisfactory recovery of oleuropein was achieved after a single salting-out CPE process. The use of sulfate salts allows the separation of polyphenols at ambient temperatures, which makes the proposed method an attractive alternative to conventional extraction techniques.

The low water content, as well as the low diluted oxygen in the surfactant-rich phase seems to be the main parameters that led to entrapped oleuropein showing a three times higher heat tolerance compared to the free one. In addition, the separation of polyphenols from olive leaf extract by salting-out CPE did not affect their antioxidant activity.

In conclusion, the results presented in this paper are expected to contribute positively to the development of effective, fast, technically simply and low-cost separation processes for polyphenols from vegetable sources for analytical purposes, as well as for the production of natural antioxidants free of any toxic residue, which can be used in food and the pharmaceutical industry.
